# Congress of Universities

**Published:** 1920-11-20

**Authors:** 


### Congress of Universities.

THE first Congress of Universities of the Empire was held in London in 1912, when all, to the number of fifty-three, were represented, in most cases by their executive heads, together with several members of their professoriate. The Universities Bureau was an outcome of this Congress. To it was entrusted the summoning of future Congresses at intervals of five years. In 1917 this intention was frustrated by the war. Transport difficulties rendered it undesirable to attempt to carry it into effect during the present year. It has now been arranged that the second Congress shall be held next summer. Dr. Alex Hill, who took over the organisation of the first Congress after the death of Dr. R. D. Roberts, who had laid its foundations, and has acted as secretary to the Bureau since its institution, is engaged in its promotion. The number of universities in the Empire has now increased to fifty-eight, and all, it is anticipated, will make a point of sending delegates to this great Council on Higher Education. With great generosity the University of Oxford has invited all members of the Congress to be its guests from July 5 to July 8. The Chancellor of the University, Lord Curzon, will preside over the morning session on July 5, and Mr. A. J. Balfour, Chancellor of the University of Cambridge, will preside in the afternoon. On the preceding day the Congress will assemble in London for certain ceremonial functions and entertainments, of which the programme will be announced at a later date.

